# Crystal structure of 1,1′-{(1*E*,1′*E*)-[4,4′-(9*H*-fluorene-9,9-di­yl)bis­(4,1-phenyl­ene)]bis­(aza­nylyl­idene)bis(methanylyl­idene)}bis­(naphthalen-2-ol) di­chloro­benzene monosolvate

**DOI:** 10.1107/S2056989020012104

**Published:** 2020-09-04

**Authors:** Ayalew Wodajo, Alexander G. Tskhovrebov, Tuan Anh Le, Alexey S. Kubasov, Maria M. Grishina, Oleg N. Krutius, Victor N. Khrustalev

**Affiliations:** a College of Natural and Computational Sciences, University of Gondar, 196 Gondar, Ethiopia; b N. N. Semenov Federal Research Center for Chemical Physics, Russian Academy of Sciences, Ul. Kosygina 4, Moscow, Russian Federation; c Peoples Friendship University of Russia, 6 Miklukho-Maklaya Street, Moscow, 117198, Russian Federation; d Faculty of Chemistry, VNU University of Science, 334 Nguyen Trai, Thanh Xuan, Hanoi, Vietnam; e Kurnakov Institute of General and Inorganic Chemistry, Russian Academy of Sciences, Leninsky Prosp. 31, Moscow, Russian Federation

**Keywords:** azomethines, imines, hydrogen bonding, weak inter­actions, excited-state intra­molecular proton transfer, crystal structure

## Abstract

The bis­(anil) mol­ecule of the title compound, C_47_H_32_N_2_O_2_·C_6_H_4_Cl_2_, contains two anil fragments in the enol–enol form, exhibiting intra­molecular O—H⋯N hydrogen bonds. The two hy­droxy­naphthalene ring systems are approximately parallel to each other with a dihedral angle of 4.67 (8)° between them, and each ring system makes a large dihedral angle [55.11 (11) and 48.50 (10)°] with the adjacent benzene ring. In the crystal, the bis­(anil) mol­ecules form an inversion dimer by a pair of weak C—H⋯O inter­actions. The dimers arrange in a one-dimensional column along the *b* axis *via* another C—H⋯O inter­action and a π–π stacking inter­action between the hy­droxy­naphthalene ring system with a centroid–centroid distance of 3.6562 (16) Å. The solvent 1,2-di­chloro­benzene mol­ecules are located between the dimers and bind neighbouring columns by weak C—H⋯Cl inter­actions. Theoretical prediction of potential biological activities was performed, which suggested that the title anil compound can exhibit histone de­acetyl­ase SIRT2, histone de­acetyl­ase class III and histone de­acetyl­ase SIRT1 activities, and will act as inhibitor to aspulvinone di­methyl­allyl­transferase, de­hydro-l-gulonate deca­rboxylase and gluta­thione thiol­esterase.

## Abstract 2

A novel bis­(anil) compound was synthesized and structurally characterized. Theoretical calculations suggested that the new bis­(hy­droxy­imine) will exhibit histone de­acetyl­ase SIRT2, histone de­acetyl­ase class III and histone de­acetyl­ase SIRT1 activities, and will act as inhibitor to aspulvinone di­methyl­allyl­transferase, de­hydro-l-gulonate deca­rboxylase and gluta­thione thiol­esterase.

## Chemical context   

Schiff bases formed by the condensation of salicyl­aldehydes with amines are also known as anils. They often exhibit potent anti­bacterial, anti­proliferative and anti­toxic properties (Williams, 1972 ▸). In addition, they are an important class of ligands, which are widely used in inorganic and coordination chemistry (Devi *et al.*, 2019 ▸). Non-coordinating anils undergo excited-state intra­molecular proton transfer (ESIPT), which make them attractive objects for photophysical investigations (Minkin *et al.*, 2011 ▸; Cohen & Schmidt, 1962 ▸). Their colours and proton-transfer equilibrium is greatly dependent on the substituents in the core (Sliwa *et al.*, 2009 ▸, 2010 ▸). Here we describe a crystal structure of the title compound, which was synthesized by the condensation between 4,4′-(9*H*-fluorene-9,9-di­yl)dianiline and two equivalents of 2-hy­droxy-1-naphthaldehyde. According to the *PASS* program – computer prediction of biological activities (Filimonov *et al.*, 2014 ▸), the title compound will exhibit histone de­acetyl­ase SIRT2, histone de­acetyl­ase class III and histone de­acetyl­ase SIRT1 (91, 86 and 73%, respectively), and will act inhibitor of enzymes, such as aspulvinone di­methyl­allyl­transferase (81% probability), de­hydro-l-gulonate deca­rboxylase (75%) and gluta­thione thiol­esterase (71%).
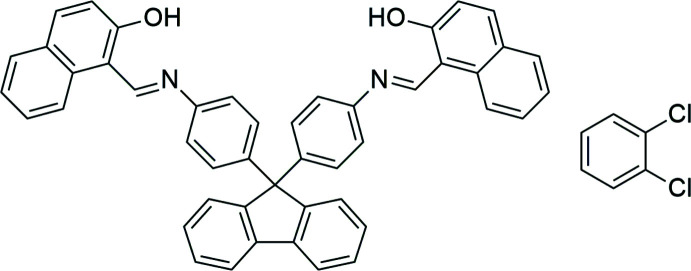


## Structural commentary   

**Figure 1:**
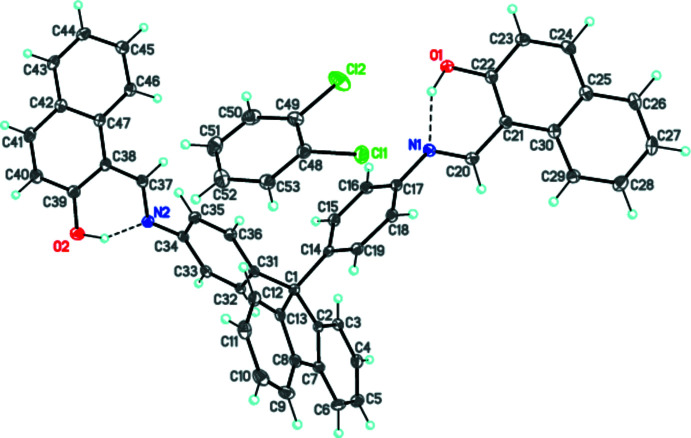
Mol­ecular structure of the title compound. Displacement ellipsoids are shown at the 50% probability level. H atoms are presented as small spheres of arbitrary radius. Dashed lines indicate the intra­molecular O—H⋯N hydrogen bonds (Table 1 ▸).

In the title bis­(anil) mol­ecule, two hy­droxy­naphthalene ring systems are approximately parallel to each other with a dihedral angle of 4.67 (8)° between them (Fig. 1 ▸). The 9*H*-fluorene ring system (C1–C13) forms large dihedral angles of 78.80 (10) and 61.41 (9)°, respectively, with the benzene C14–C19 and C31–C36 rings. Each hy­droxy­naphthalene ring system also forms a large dihedral angle with the adjacent benzene ring [55.11 (11)° between the C21–C30 ring system and the C14–C19 ring, and 48.50 (10)° between the C38–C47 ring system and the C31–C36 ring]. Both fragments of the hy­droxy­naphthalene Schiff bases are in the enol form, forming intra­molecular O—H⋯N hydrogen bonds (Table 1 ▸).

## Supra­molecular features   

**Figure 2:**
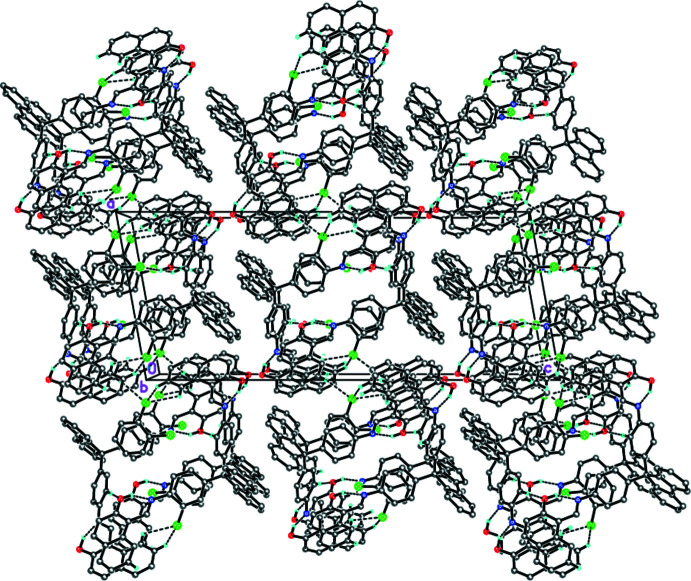
A packing diagram of the title compound. Dashed lines indicate the intra­molecular O—H⋯N hydrogen bonds and the inter­molecular C—H⋯O and C—H⋯Cl inter­actions.

In the crystal, the bis­(anil) mol­ecules form an inversion dimer *via* a pair of weak C—H⋯O inter­actions (C3—H3⋯O1^i^; symmetry code given in Table 1 ▸). The dimers form a 1D column along the *b* axis through a C—H⋯O (C35—H35⋯O1^ii^; Table 1 ▸) and a π–π stacking inter­action between the hydroxyl naphthalene ring systems with a centroid-centroid distance of 3.6562 (16) Å (*Cg*1⋯*Cg*2^ii^; *Cg*1 and *Cg*2 are the centroids of C21–C30 and C38–C47 ring systems, respectively). Di­chloro­benzene mol­ecules are located between the dimers and bind the neighboring columns by weak C—H⋯Cl inter­actions (Table 1 ▸ and Fig. 2 ▸).

## Database survey   

A search of the Cambridge Structural Database (CSD version 5.41, update of March, 2020; Groom *et al.*, 2016 ▸) revealed the existence of several structurally similar bis-hy­droxy­imines derivatives. All of them were prepared *via* the condensation of the corresponding di­amine and an appropriate hy­droxy­aldehyde (Elmali *et al.*, 1995 ▸; Blagus & Kaitner, 2011 ▸; Popović *et al.*, 2001 ▸; Meng *et al.*, 2008 ▸; Wang *et al.*, 2016 ▸; Han *et al.*, 2015 ▸). Inter­estingly, although keto–enol tautamerization is a well-established phenomenon for such systems, the majority of known bis-hy­droxy­imines exist in enol-enol forms, except the one reported by Popović *et al.* (2001 ▸).

## Synthesis and crystallization   

The compound was obtained by the condensation between 2-hy­droxy-1-naphthaldehyde and 4,4′-(9*H*-fluorene-9,9-di­yl)dianiline according to the literature (Elhusseiny *et al.*, 2015 ▸; Kundu *et al.*, 2015 ▸). Single crystals suitable for the X-ray analysis were obtained by the slow evaporation of a saturated 1,2-di­chloro­benzene solution.

## Refinement   

Crystal data, details of data collection, and results of structure refinement are summarized in Table 2 ▸. All C-bound H atoms were placed in calculated positions (C—H = 0.95 Å) and refined using a riding model [*U*_iso_(H) = 1.2*U*_eq_(C)], while the H atoms of the OH groups were localized in a difference-Fourier map and refined with *U*_iso_(H) = 1.5*U*_eq_(O).

## Supplementary Material

Crystal structure: contains datablock(s) general, I. DOI: 10.1107/S2056989020012104/is5554sup1.cif

Structure factors: contains datablock(s) I. DOI: 10.1107/S2056989020012104/is5554Isup2.hkl

CCDC reference: 2019265

Additional supporting information:  crystallographic information; 3D view; checkCIF report

## Figures and Tables

**Table 1:** Hydrogen-bond geometry (Å, °)

*D*—H⋯*A*	*D*—H	H⋯*A*	*D*⋯*A*	*D*—H⋯*A*
O1—H1⋯N1	0.87 (3)	1.75 (3)	2.526 (3)	148 (3)
O2—H2⋯N2	1.00 (4)	1.63 (4)	2.558 (3)	152 (3)
C3—H3⋯O1^i^	0.95	2.58	3.461 (3)	155
C35—H35⋯O1^ii^	0.95	2.44	3.389 (3)	178
C28—H28⋯Cl1^iii^	0.95	2.86	3.770 (3)	161
C45—H45⋯Cl1^ii^	0.95	2.86	3.457 (3)	122
C46—H46⋯Cl1^ii^	0.95	2.88	3.465 (3)	121

Symmetry codes: (i) 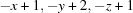; (ii) 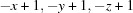; (iii) 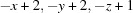.

**Table 2:** Experimental details

Crystal data	
Chemical formula	C_47_H_32_N_2_O_2_·C_6_H_4_Cl_2_
*M*_r_	803.74
Crystal system, space group	Monoclinic, *P*2_1_/*c*
Temperature (K)	100
*a*, *b*, *c* (Å)	13.3070 (6), 9.1782 (4), 32.2298 (16)
β (°)	100.251 (2)
*V* (Å^3^)	3873.5 (3)
*Z*	4
Radiation type	Mo *K*α
μ (mm^−1^)	0.22
Crystal size (mm)	0.8 × 0.4 × 0.1

Data collection	
Diffractometer	Bruker APEXII CCD
Absorption correction	Multi-scan (*SADABS*; Bruker, 2016 ▸)
*T*_min_, *T*_max_	0.596, 0.746
No. of measured, independent and observed [*I* > 2σ(*I*)] reflections	18958, 7377, 5542
*R*_int_	0.036
(sin θ/λ)_max_ (Å^−1^)	0.617

Refinement	
*R*[*F* ^2^ > 2σ(*F* ^2^)], *wR*(*F* ^2^), *S*	0.053, 0.140, 1.03
No. of reflections	7377
No. of parameters	538
H-atom treatment	H atoms treated by a mixture of independent and constrained refinement
Δρ_max_, Δρ_min_ (e Å^−3^)	0.33, −0.41

Computer programs: *APEX3* (Bruker, 2018 ▸), *SAINT* (Bruker, 2016 ▸), *SHELXT* (Sheldrick, 2015*a* ▸), *SHELXL2018/3* (Sheldrick, 2015*b* ▸) and *OLEX2* (Dolomanov *et al.*, 2009 ▸).

## supplementary crystallographic information

### Crystal data

**Table:**

C_47_H_32_N_2_O_2_·C_6_H_4_Cl_2_	*F*(000) = 1672
*M**_r_* = 803.74	*D*_x_ = 1.378 Mg m^−^^3^
Monoclinic, *P*2_1_/*c*	Mo *K*α radiation, λ = 0.71073 Å
*a* = 13.3070 (6) Å	Cell parameters from 6276 reflections
*b* = 9.1782 (4) Å	θ = 2.6–29.6°
*c* = 32.2298 (16) Å	µ = 0.22 mm^−^^1^
β = 100.251 (2)°	*T* = 100 K
*V* = 3873.5 (3) Å^3^	Plate, yellow
*Z* = 4	0.8 × 0.4 × 0.1 mm

### Data collection

**Table:**

Bruker APEXII CCD diffractometer	5542 reflections with *I* > 2σ(*I*)
φ and ω scans	*R*_int_ = 0.036
Absorption correction: multi-scan (SADABS; Bruker, 2016)	θ_max_ = 26.0°, θ_min_ = 1.8°
*T*_min_ = 0.596, *T*_max_ = 0.746	*h* = −16→16
18958 measured reflections	*k* = −10→11
7377 independent reflections	*l* = −33→39

### Refinement

**Table:**

Refinement on *F*^2^	0 restraints
Least-squares matrix: full	Hydrogen site location: mixed
*R*[*F*^2^ > 2σ(*F*^2^)] = 0.053	H atoms treated by a mixture of independent and constrained refinement
*wR*(*F*^2^) = 0.140	*w* = 1/[σ^2^(*F*_o_^2^) + (0.0606*P*)^2^ + 3.6798*P*] where *P* = (*F*_o_^2^ + 2*F*_c_^2^)/3
*S* = 1.03	(Δ/σ)_max_ = 0.001
7377 reflections	Δρ_max_ = 0.33 e Å^−^^3^
538 parameters	Δρ_min_ = −0.41 e Å^−^^3^

### Special details

**Table:**

Geometry. All esds (except the esd in the dihedral angle between two l.s. planes) are estimated using the full covariance matrix. The cell esds are taken into account individually in the estimation of esds in distances, angles and torsion angles; correlations between esds in cell parameters are only used when they are defined by crystal symmetry. An approximate (isotropic) treatment of cell esds is used for estimating esds involving l.s. planes.

### Fractional atomic coordinates and isotropic or equivalent isotropic displacement parameters (Å^2^)

**Table:**

	*x*	*y*	*z*	*U*_iso_*/*U*_eq_	
Cl1	0.86399 (5)	0.50325 (8)	0.48868 (2)	0.03159 (18)	
Cl2	0.67342 (7)	0.49018 (11)	0.53468 (2)	0.0506 (2)	
C48	0.7535 (2)	0.4140 (3)	0.46574 (8)	0.0240 (6)	
C49	0.6694 (2)	0.4093 (3)	0.48571 (8)	0.0293 (6)	
C50	0.5823 (2)	0.3367 (3)	0.46676 (10)	0.0363 (7)	
H50	0.524224	0.334047	0.480129	0.044*	
C51	0.5794 (2)	0.2680 (3)	0.42838 (10)	0.0374 (7)	
H51	0.519534	0.217721	0.415533	0.045*	
C52	0.6634 (2)	0.2723 (3)	0.40872 (10)	0.0341 (7)	
H52	0.661665	0.223847	0.382545	0.041*	
C53	0.7503 (2)	0.3472 (3)	0.42716 (8)	0.0275 (6)	
H53	0.807552	0.352531	0.413313	0.033*	
O1	0.67650 (15)	0.8293 (2)	0.59900 (6)	0.0254 (4)	
H1	0.655 (2)	0.836 (3)	0.5719 (10)	0.038*	
O2	0.00869 (17)	0.3629 (2)	0.27222 (6)	0.0346 (5)	
H2	0.071 (3)	0.422 (4)	0.2835 (11)	0.052*	
N1	0.67572 (17)	0.9057 (2)	0.52365 (6)	0.0218 (5)	
N2	0.15609 (16)	0.4833 (2)	0.32226 (7)	0.0224 (5)	
C1	0.54205 (19)	0.7866 (3)	0.34681 (7)	0.0185 (5)	
C2	0.53815 (19)	0.9371 (3)	0.32573 (7)	0.0178 (5)	
C3	0.4827 (2)	1.0591 (3)	0.33307 (8)	0.0217 (6)	
H3	0.439149	1.056724	0.353428	0.026*	
C4	0.4920 (2)	1.1856 (3)	0.31005 (8)	0.0255 (6)	
H4	0.454262	1.270090	0.314727	0.031*	
C5	0.5559 (2)	1.1893 (3)	0.28034 (8)	0.0285 (6)	
H5	0.561040	1.275926	0.264729	0.034*	
C6	0.6123 (2)	1.0679 (3)	0.27327 (8)	0.0261 (6)	
H6	0.656020	1.070707	0.252987	0.031*	
C7	0.60383 (19)	0.9421 (3)	0.29627 (7)	0.0208 (5)	
C8	0.65444 (19)	0.8007 (3)	0.29556 (8)	0.0213 (5)	
C9	0.7264 (2)	0.7520 (3)	0.27193 (8)	0.0274 (6)	
H9	0.750623	0.815367	0.252576	0.033*	
C10	0.7617 (2)	0.6106 (3)	0.27713 (9)	0.0316 (7)	
H10	0.810846	0.576477	0.261388	0.038*	
C11	0.7257 (2)	0.5180 (3)	0.30522 (9)	0.0300 (6)	
H11	0.750040	0.420553	0.308222	0.036*	
C12	0.6548 (2)	0.5654 (3)	0.32899 (8)	0.0245 (6)	
H12	0.630758	0.501280	0.348205	0.029*	
C13	0.61944 (19)	0.7073 (3)	0.32437 (7)	0.0202 (5)	
C14	0.58069 (19)	0.8082 (3)	0.39440 (7)	0.0187 (5)	
C15	0.5104 (2)	0.8411 (3)	0.42059 (8)	0.0219 (6)	
H15	0.439472	0.842256	0.409218	0.026*	
C16	0.5432 (2)	0.8722 (3)	0.46297 (8)	0.0215 (5)	
H16	0.494462	0.891713	0.480497	0.026*	
C17	0.64629 (19)	0.8750 (3)	0.47993 (8)	0.0194 (5)	
C18	0.71678 (19)	0.8396 (3)	0.45441 (8)	0.0206 (5)	
H18	0.787676	0.838484	0.465863	0.025*	
C19	0.6834 (2)	0.8059 (3)	0.41211 (8)	0.0212 (5)	
H19	0.732035	0.780775	0.395037	0.025*	
C20	0.75022 (19)	0.9948 (3)	0.53695 (8)	0.0206 (5)	
H20	0.780899	1.046811	0.516987	0.025*	
C21	0.78777 (19)	1.0168 (3)	0.58159 (8)	0.0199 (5)	
C22	0.75129 (19)	0.9278 (3)	0.61090 (8)	0.0201 (5)	
C23	0.7938 (2)	0.9344 (3)	0.65415 (8)	0.0233 (6)	
H23	0.767240	0.874321	0.673656	0.028*	
C24	0.8724 (2)	1.0262 (3)	0.66798 (8)	0.0240 (6)	
H24	0.901129	1.027626	0.697156	0.029*	
C25	0.9131 (2)	1.1202 (3)	0.64022 (8)	0.0227 (6)	
C26	0.9951 (2)	1.2152 (3)	0.65491 (9)	0.0273 (6)	
H26	1.024204	1.215786	0.684048	0.033*	
C27	1.0332 (2)	1.3061 (3)	0.62817 (9)	0.0300 (6)	
H27	1.089811	1.367247	0.638394	0.036*	
C28	0.9882 (2)	1.3087 (3)	0.58537 (9)	0.0311 (7)	
H28	1.013243	1.374462	0.566853	0.037*	
C29	0.9088 (2)	1.2179 (3)	0.56990 (8)	0.0254 (6)	
H29	0.879911	1.221614	0.540761	0.031*	
C30	0.86878 (19)	1.1184 (3)	0.59640 (8)	0.0203 (5)	
C31	0.43890 (19)	0.7081 (3)	0.33971 (7)	0.0179 (5)	
C32	0.35105 (19)	0.7645 (3)	0.31484 (7)	0.0190 (5)	
H32	0.354567	0.855460	0.301084	0.023*	
C33	0.2587 (2)	0.6909 (3)	0.30973 (7)	0.0201 (5)	
H33	0.200010	0.731800	0.292563	0.024*	
C34	0.2513 (2)	0.5582 (3)	0.32948 (7)	0.0204 (5)	
C35	0.3380 (2)	0.4996 (3)	0.35461 (8)	0.0222 (6)	
H35	0.334273	0.408341	0.368175	0.027*	
C36	0.4297 (2)	0.5747 (3)	0.35972 (8)	0.0210 (5)	
H36	0.487955	0.534482	0.377314	0.025*	
C37	0.1256 (2)	0.4184 (3)	0.35336 (8)	0.0213 (5)	
H37	0.163220	0.431205	0.381049	0.026*	
C38	0.03563 (19)	0.3267 (3)	0.34722 (8)	0.0206 (5)	
C39	−0.0164 (2)	0.2972 (3)	0.30650 (8)	0.0251 (6)	
C40	−0.0959 (2)	0.1940 (3)	0.29929 (9)	0.0313 (7)	
H40	−0.130203	0.175740	0.271316	0.038*	
C41	−0.1241 (2)	0.1202 (3)	0.33190 (9)	0.0291 (6)	
H41	−0.177120	0.049838	0.326410	0.035*	
C42	−0.07517 (19)	0.1470 (3)	0.37423 (8)	0.0226 (6)	
C43	−0.1041 (2)	0.0681 (3)	0.40780 (9)	0.0261 (6)	
H43	−0.155966	−0.003853	0.402031	0.031*	
C44	−0.0582 (2)	0.0942 (3)	0.44851 (9)	0.0270 (6)	
H44	−0.077970	0.040511	0.470940	0.032*	
C45	0.0181 (2)	0.2004 (3)	0.45698 (8)	0.0251 (6)	
H45	0.049100	0.219549	0.485344	0.030*	
C46	0.04883 (19)	0.2774 (3)	0.42492 (8)	0.0224 (6)	
H46	0.101119	0.348534	0.431445	0.027*	
C47	0.00390 (19)	0.2526 (3)	0.38229 (8)	0.0197 (5)	

### Atomic displacement parameters (Å^2^)

**Table:**

	*U*^11^	*U*^22^	*U*^33^	*U*^12^	*U*^13^	*U*^23^
Cl1	0.0293 (4)	0.0315 (4)	0.0332 (4)	−0.0064 (3)	0.0033 (3)	−0.0046 (3)
Cl2	0.0444 (5)	0.0794 (6)	0.0306 (4)	0.0072 (5)	0.0134 (3)	−0.0107 (4)
C48	0.0236 (14)	0.0201 (13)	0.0271 (14)	0.0005 (11)	0.0013 (11)	0.0024 (11)
C49	0.0308 (16)	0.0329 (15)	0.0242 (14)	0.0011 (13)	0.0055 (12)	0.0021 (12)
C50	0.0237 (16)	0.0461 (18)	0.0400 (17)	−0.0019 (14)	0.0084 (13)	0.0111 (15)
C51	0.0247 (16)	0.0341 (16)	0.0491 (19)	−0.0065 (13)	−0.0049 (14)	0.0022 (14)
C52	0.0336 (17)	0.0318 (16)	0.0348 (16)	0.0027 (14)	0.0001 (13)	−0.0051 (13)
C53	0.0285 (15)	0.0280 (15)	0.0261 (14)	0.0018 (12)	0.0050 (12)	0.0011 (12)
O1	0.0310 (11)	0.0256 (10)	0.0199 (9)	−0.0065 (8)	0.0056 (8)	0.0014 (8)
O2	0.0384 (12)	0.0408 (12)	0.0223 (10)	−0.0112 (10)	−0.0008 (9)	0.0044 (9)
N1	0.0253 (12)	0.0217 (11)	0.0183 (11)	−0.0013 (10)	0.0034 (9)	−0.0008 (9)
N2	0.0234 (12)	0.0219 (11)	0.0229 (11)	−0.0033 (9)	0.0070 (9)	−0.0010 (9)
C1	0.0205 (13)	0.0176 (12)	0.0180 (12)	−0.0020 (10)	0.0051 (10)	−0.0012 (10)
C2	0.0178 (13)	0.0199 (12)	0.0145 (11)	−0.0048 (10)	−0.0005 (10)	0.0005 (10)
C3	0.0250 (14)	0.0224 (13)	0.0174 (12)	−0.0040 (11)	0.0029 (11)	−0.0021 (10)
C4	0.0277 (15)	0.0210 (13)	0.0254 (14)	−0.0003 (12)	−0.0022 (11)	0.0007 (11)
C5	0.0307 (16)	0.0274 (14)	0.0243 (14)	−0.0057 (13)	−0.0037 (12)	0.0096 (11)
C6	0.0235 (15)	0.0335 (15)	0.0215 (13)	−0.0043 (12)	0.0049 (11)	0.0082 (12)
C7	0.0179 (13)	0.0279 (14)	0.0157 (12)	−0.0054 (11)	0.0007 (10)	0.0010 (10)
C8	0.0165 (13)	0.0296 (14)	0.0171 (12)	−0.0036 (11)	0.0010 (10)	−0.0032 (11)
C9	0.0220 (14)	0.0409 (16)	0.0201 (13)	−0.0004 (13)	0.0058 (11)	0.0009 (12)
C10	0.0266 (15)	0.0431 (17)	0.0260 (14)	0.0038 (13)	0.0075 (12)	−0.0091 (13)
C11	0.0270 (15)	0.0269 (15)	0.0356 (15)	0.0006 (12)	0.0044 (13)	−0.0106 (12)
C12	0.0242 (14)	0.0223 (13)	0.0274 (14)	−0.0034 (11)	0.0055 (11)	−0.0043 (11)
C13	0.0187 (13)	0.0242 (13)	0.0171 (12)	−0.0058 (11)	0.0015 (10)	−0.0049 (10)
C14	0.0252 (14)	0.0128 (11)	0.0185 (12)	−0.0045 (10)	0.0047 (10)	0.0014 (10)
C15	0.0203 (14)	0.0215 (13)	0.0238 (13)	−0.0022 (11)	0.0037 (11)	−0.0011 (11)
C16	0.0220 (14)	0.0228 (13)	0.0213 (13)	−0.0001 (11)	0.0080 (11)	−0.0011 (10)
C17	0.0213 (14)	0.0178 (12)	0.0187 (12)	−0.0014 (11)	0.0021 (10)	0.0018 (10)
C18	0.0151 (13)	0.0229 (13)	0.0225 (13)	−0.0024 (11)	−0.0004 (10)	0.0001 (10)
C19	0.0233 (14)	0.0200 (13)	0.0212 (13)	−0.0021 (11)	0.0067 (11)	0.0003 (10)
C20	0.0215 (14)	0.0197 (12)	0.0214 (12)	0.0023 (11)	0.0060 (10)	0.0018 (10)
C21	0.0211 (13)	0.0192 (12)	0.0193 (12)	0.0022 (11)	0.0037 (10)	−0.0015 (10)
C22	0.0210 (14)	0.0171 (12)	0.0225 (13)	0.0032 (11)	0.0046 (11)	−0.0020 (10)
C23	0.0295 (15)	0.0212 (13)	0.0207 (13)	0.0042 (12)	0.0091 (11)	−0.0003 (10)
C24	0.0279 (15)	0.0263 (14)	0.0175 (12)	0.0076 (12)	0.0034 (11)	−0.0037 (11)
C25	0.0220 (14)	0.0221 (13)	0.0240 (13)	0.0047 (11)	0.0042 (11)	−0.0051 (11)
C26	0.0247 (15)	0.0276 (14)	0.0272 (14)	0.0028 (12)	−0.0016 (12)	−0.0082 (12)
C27	0.0224 (15)	0.0267 (14)	0.0387 (16)	−0.0046 (12)	−0.0001 (12)	−0.0099 (13)
C28	0.0336 (17)	0.0250 (14)	0.0368 (16)	−0.0035 (13)	0.0120 (13)	−0.0006 (12)
C29	0.0285 (15)	0.0240 (14)	0.0239 (13)	−0.0012 (12)	0.0050 (11)	0.0002 (11)
C30	0.0179 (13)	0.0189 (12)	0.0240 (13)	0.0034 (11)	0.0037 (10)	−0.0028 (10)
C31	0.0210 (13)	0.0174 (12)	0.0156 (12)	0.0007 (10)	0.0044 (10)	−0.0041 (10)
C32	0.0255 (14)	0.0177 (12)	0.0145 (11)	−0.0022 (11)	0.0056 (10)	−0.0029 (10)
C33	0.0208 (13)	0.0226 (13)	0.0169 (12)	0.0014 (11)	0.0034 (10)	−0.0038 (10)
C34	0.0229 (14)	0.0242 (13)	0.0157 (12)	−0.0050 (11)	0.0081 (10)	−0.0055 (10)
C35	0.0262 (14)	0.0197 (13)	0.0224 (13)	−0.0015 (11)	0.0086 (11)	−0.0003 (11)
C36	0.0210 (14)	0.0218 (13)	0.0204 (13)	−0.0003 (11)	0.0041 (10)	0.0013 (10)
C37	0.0217 (14)	0.0218 (13)	0.0209 (13)	0.0007 (11)	0.0047 (11)	−0.0031 (10)
C38	0.0190 (13)	0.0171 (12)	0.0256 (13)	0.0008 (11)	0.0040 (10)	−0.0011 (10)
C39	0.0236 (14)	0.0264 (14)	0.0241 (13)	−0.0006 (12)	0.0012 (11)	0.0029 (11)
C40	0.0294 (16)	0.0337 (16)	0.0267 (14)	−0.0056 (13)	−0.0061 (12)	0.0005 (12)
C41	0.0228 (15)	0.0262 (14)	0.0358 (16)	−0.0067 (12)	−0.0020 (12)	−0.0009 (12)
C42	0.0186 (13)	0.0202 (13)	0.0287 (14)	0.0032 (11)	0.0035 (11)	−0.0007 (11)
C43	0.0200 (14)	0.0207 (13)	0.0384 (16)	−0.0029 (11)	0.0071 (12)	0.0008 (12)
C44	0.0282 (15)	0.0239 (14)	0.0312 (15)	0.0008 (12)	0.0116 (12)	0.0045 (12)
C45	0.0244 (14)	0.0265 (14)	0.0247 (14)	0.0032 (12)	0.0051 (11)	−0.0003 (11)
C46	0.0178 (13)	0.0215 (13)	0.0285 (14)	−0.0008 (11)	0.0057 (11)	−0.0027 (11)
C47	0.0150 (13)	0.0190 (12)	0.0254 (13)	0.0017 (10)	0.0044 (10)	−0.0021 (10)

### Geometric parameters (Å, º)

**Table:**

Cl1—C48	1.730 (3)	C18—H18	0.9500
Cl2—C49	1.736 (3)	C18—C19	1.392 (4)
C48—C49	1.387 (4)	C19—H19	0.9500
C48—C53	1.380 (4)	C20—H20	0.9500
C49—C50	1.382 (4)	C20—C21	1.451 (3)
C50—H50	0.9500	C21—C22	1.399 (3)
C50—C51	1.383 (4)	C21—C30	1.441 (4)
C51—H51	0.9500	C22—C23	1.409 (4)
C51—C52	1.380 (4)	C23—H23	0.9500
C52—H52	0.9500	C23—C24	1.356 (4)
C52—C53	1.385 (4)	C24—H24	0.9500
C53—H53	0.9500	C24—C25	1.418 (4)
O1—H1	0.87 (3)	C25—C26	1.411 (4)
O1—C22	1.349 (3)	C25—C30	1.430 (4)
O2—H2	1.00 (4)	C26—H26	0.9500
O2—C39	1.351 (3)	C26—C27	1.361 (4)
N1—C17	1.422 (3)	C27—H27	0.9500
N1—C20	1.298 (3)	C27—C28	1.403 (4)
N2—C34	1.424 (3)	C28—H28	0.9500
N2—C37	1.292 (3)	C28—C29	1.368 (4)
C1—C2	1.537 (3)	C29—H29	0.9500
C1—C13	1.542 (3)	C29—C30	1.418 (4)
C1—C14	1.541 (3)	C31—C32	1.394 (4)
C1—C31	1.531 (3)	C31—C36	1.400 (3)
C2—C3	1.384 (4)	C32—H32	0.9500
C2—C7	1.401 (3)	C32—C33	1.386 (4)
C3—H3	0.9500	C33—H33	0.9500
C3—C4	1.395 (4)	C33—C34	1.386 (4)
C4—H4	0.9500	C34—C35	1.395 (4)
C4—C5	1.390 (4)	C35—H35	0.9500
C5—H5	0.9500	C35—C36	1.385 (4)
C5—C6	1.384 (4)	C36—H36	0.9500
C6—H6	0.9500	C37—H37	0.9500
C6—C7	1.388 (4)	C37—C38	1.447 (4)
C7—C8	1.464 (4)	C38—C39	1.397 (4)
C8—C9	1.399 (4)	C38—C47	1.445 (4)
C8—C13	1.403 (4)	C39—C40	1.409 (4)
C9—H9	0.9500	C40—H40	0.9500
C9—C10	1.380 (4)	C40—C41	1.358 (4)
C10—H10	0.9500	C41—H41	0.9500
C10—C11	1.388 (4)	C41—C42	1.425 (4)
C11—H11	0.9500	C42—C43	1.411 (4)
C11—C12	1.387 (4)	C42—C47	1.420 (4)
C12—H12	0.9500	C43—H43	0.9500
C12—C13	1.383 (4)	C43—C44	1.366 (4)
C14—C15	1.400 (3)	C44—H44	0.9500
C14—C19	1.384 (4)	C44—C45	1.399 (4)
C15—H15	0.9500	C45—H45	0.9500
C15—C16	1.388 (4)	C45—C46	1.373 (4)
C16—H16	0.9500	C46—H46	0.9500
C16—C17	1.383 (4)	C46—C47	1.416 (4)
C17—C18	1.392 (4)		
			
C49—C48—Cl1	120.7 (2)	N1—C20—C21	121.6 (2)
C53—C48—Cl1	118.9 (2)	C21—C20—H20	119.2
C53—C48—C49	120.4 (3)	C22—C21—C20	119.2 (2)
C48—C49—Cl2	120.8 (2)	C22—C21—C30	118.9 (2)
C50—C49—Cl2	119.7 (2)	C30—C21—C20	121.6 (2)
C50—C49—C48	119.5 (3)	O1—C22—C21	121.8 (2)
C49—C50—H50	119.9	O1—C22—C23	117.1 (2)
C49—C50—C51	120.2 (3)	C21—C22—C23	121.1 (2)
C51—C50—H50	119.9	C22—C23—H23	119.9
C50—C51—H51	119.9	C24—C23—C22	120.1 (2)
C52—C51—C50	120.1 (3)	C24—C23—H23	119.9
C52—C51—H51	119.9	C23—C24—H24	119.0
C51—C52—H52	120.0	C23—C24—C25	122.0 (2)
C51—C52—C53	120.0 (3)	C25—C24—H24	119.0
C53—C52—H52	120.0	C24—C25—C30	118.6 (2)
C48—C53—C52	119.8 (3)	C26—C25—C24	121.6 (2)
C48—C53—H53	120.1	C26—C25—C30	119.8 (2)
C52—C53—H53	120.1	C25—C26—H26	119.4
C22—O1—H1	109 (2)	C27—C26—C25	121.3 (3)
C39—O2—H2	104.5 (19)	C27—C26—H26	119.4
C20—N1—C17	120.8 (2)	C26—C27—H27	120.3
C37—N2—C34	119.3 (2)	C26—C27—C28	119.4 (3)
C2—C1—C13	100.94 (19)	C28—C27—H27	120.3
C2—C1—C14	107.63 (19)	C27—C28—H28	119.5
C14—C1—C13	113.3 (2)	C29—C28—C27	121.0 (3)
C31—C1—C2	113.5 (2)	C29—C28—H28	119.5
C31—C1—C13	111.35 (19)	C28—C29—H29	119.3
C31—C1—C14	109.93 (19)	C28—C29—C30	121.4 (3)
C3—C2—C1	129.0 (2)	C30—C29—H29	119.3
C3—C2—C7	120.4 (2)	C25—C30—C21	119.2 (2)
C7—C2—C1	110.6 (2)	C29—C30—C21	123.8 (2)
C2—C3—H3	120.6	C29—C30—C25	117.0 (2)
C2—C3—C4	118.8 (2)	C32—C31—C1	123.3 (2)
C4—C3—H3	120.6	C32—C31—C36	117.2 (2)
C3—C4—H4	119.7	C36—C31—C1	119.5 (2)
C5—C4—C3	120.6 (3)	C31—C32—H32	119.3
C5—C4—H4	119.7	C33—C32—C31	121.5 (2)
C4—C5—H5	119.7	C33—C32—H32	119.3
C6—C5—C4	120.7 (2)	C32—C33—H33	119.8
C6—C5—H5	119.7	C34—C33—C32	120.5 (2)
C5—C6—H6	120.5	C34—C33—H33	119.8
C5—C6—C7	119.0 (2)	C33—C34—N2	118.9 (2)
C7—C6—H6	120.5	C33—C34—C35	119.1 (2)
C2—C7—C8	109.0 (2)	C35—C34—N2	121.9 (2)
C6—C7—C2	120.6 (2)	C34—C35—H35	120.1
C6—C7—C8	130.4 (2)	C36—C35—C34	119.8 (2)
C9—C8—C7	130.8 (2)	C36—C35—H35	120.1
C9—C8—C13	120.3 (2)	C31—C36—H36	119.1
C13—C8—C7	108.9 (2)	C35—C36—C31	121.9 (2)
C8—C9—H9	120.5	C35—C36—H36	119.1
C10—C9—C8	119.0 (3)	N2—C37—H37	119.0
C10—C9—H9	120.5	N2—C37—C38	121.9 (2)
C9—C10—H10	119.8	C38—C37—H37	119.0
C9—C10—C11	120.4 (3)	C39—C38—C37	120.0 (2)
C11—C10—H10	119.8	C39—C38—C47	118.6 (2)
C10—C11—H11	119.5	C47—C38—C37	121.1 (2)
C10—C11—C12	121.0 (3)	O2—C39—C38	121.9 (2)
C12—C11—H11	119.5	O2—C39—C40	116.9 (2)
C11—C12—H12	120.4	C38—C39—C40	121.1 (2)
C13—C12—C11	119.2 (3)	C39—C40—H40	119.7
C13—C12—H12	120.4	C41—C40—C39	120.7 (2)
C8—C13—C1	110.5 (2)	C41—C40—H40	119.7
C12—C13—C1	129.5 (2)	C40—C41—H41	119.6
C12—C13—C8	120.0 (2)	C40—C41—C42	120.9 (3)
C15—C14—C1	119.2 (2)	C42—C41—H41	119.6
C19—C14—C1	122.6 (2)	C43—C42—C41	120.4 (2)
C19—C14—C15	118.1 (2)	C43—C42—C47	120.3 (2)
C14—C15—H15	119.6	C47—C42—C41	119.4 (2)
C16—C15—C14	120.7 (2)	C42—C43—H43	119.7
C16—C15—H15	119.6	C44—C43—C42	120.7 (2)
C15—C16—H16	119.7	C44—C43—H43	119.7
C17—C16—C15	120.6 (2)	C43—C44—H44	120.2
C17—C16—H16	119.7	C43—C44—C45	119.6 (2)
C16—C17—N1	118.3 (2)	C45—C44—H44	120.2
C16—C17—C18	119.2 (2)	C44—C45—H45	119.5
C18—C17—N1	122.4 (2)	C46—C45—C44	121.0 (2)
C17—C18—H18	120.0	C46—C45—H45	119.5
C17—C18—C19	120.0 (2)	C45—C46—H46	119.4
C19—C18—H18	120.0	C45—C46—C47	121.1 (2)
C14—C19—C18	121.4 (2)	C47—C46—H46	119.4
C14—C19—H19	119.3	C42—C47—C38	119.2 (2)
C18—C19—H19	119.3	C46—C47—C38	123.5 (2)
N1—C20—H20	119.2	C46—C47—C42	117.3 (2)
			
Cl1—C48—C49—Cl2	−1.1 (3)	C15—C16—C17—C18	−3.0 (4)
Cl1—C48—C49—C50	−179.7 (2)	C16—C17—C18—C19	1.8 (4)
Cl1—C48—C53—C52	178.5 (2)	C17—N1—C20—C21	−174.0 (2)
Cl2—C49—C50—C51	−177.9 (2)	C17—C18—C19—C14	0.7 (4)
C48—C49—C50—C51	0.7 (5)	C19—C14—C15—C16	0.7 (4)
C49—C48—C53—C52	−1.4 (4)	C20—N1—C17—C16	−136.1 (3)
C49—C50—C51—C52	−0.4 (5)	C20—N1—C17—C18	47.7 (3)
C50—C51—C52—C53	−0.9 (5)	C20—C21—C22—O1	−5.1 (4)
C51—C52—C53—C48	1.7 (4)	C20—C21—C22—C23	172.9 (2)
C53—C48—C49—Cl2	178.8 (2)	C20—C21—C30—C25	−170.8 (2)
C53—C48—C49—C50	0.2 (4)	C20—C21—C30—C29	8.7 (4)
O1—C22—C23—C24	177.0 (2)	C21—C22—C23—C24	−1.1 (4)
O2—C39—C40—C41	178.3 (3)	C22—C21—C30—C25	3.2 (4)
N1—C17—C18—C19	177.9 (2)	C22—C21—C30—C29	−177.3 (2)
N1—C20—C21—C22	7.6 (4)	C22—C23—C24—C25	1.4 (4)
N1—C20—C21—C30	−178.4 (2)	C23—C24—C25—C26	−179.9 (2)
N2—C34—C35—C36	178.1 (2)	C23—C24—C25—C30	0.6 (4)
N2—C37—C38—C39	5.0 (4)	C24—C25—C26—C27	−179.4 (3)
N2—C37—C38—C47	178.7 (2)	C24—C25—C30—C21	−2.9 (4)
C1—C2—C3—C4	179.5 (2)	C24—C25—C30—C29	177.6 (2)
C1—C2—C7—C6	179.8 (2)	C25—C26—C27—C28	2.0 (4)
C1—C2—C7—C8	0.0 (3)	C26—C25—C30—C21	177.6 (2)
C1—C14—C15—C16	−175.1 (2)	C26—C25—C30—C29	−1.9 (4)
C1—C14—C19—C18	173.7 (2)	C26—C27—C28—C29	−2.2 (4)
C1—C31—C32—C33	−179.0 (2)	C27—C28—C29—C30	0.2 (4)
C1—C31—C36—C35	179.6 (2)	C28—C29—C30—C21	−177.7 (2)
C2—C1—C13—C8	0.7 (3)	C28—C29—C30—C25	1.8 (4)
C2—C1—C13—C12	−178.8 (3)	C30—C21—C22—O1	−179.2 (2)
C2—C1—C14—C15	86.6 (3)	C30—C21—C22—C23	−1.2 (4)
C2—C1—C14—C19	−89.0 (3)	C30—C25—C26—C27	0.1 (4)
C2—C1—C31—C32	3.0 (3)	C31—C1—C2—C3	61.9 (3)
C2—C1—C31—C36	−175.3 (2)	C31—C1—C2—C7	−119.6 (2)
C2—C3—C4—C5	−0.2 (4)	C31—C1—C13—C8	121.5 (2)
C2—C7—C8—C9	−179.5 (3)	C31—C1—C13—C12	−58.1 (3)
C2—C7—C8—C13	0.5 (3)	C31—C1—C14—C15	−37.4 (3)
C3—C2—C7—C6	−1.7 (4)	C31—C1—C14—C19	147.0 (2)
C3—C2—C7—C8	178.5 (2)	C31—C32—C33—C34	0.1 (4)
C3—C4—C5—C6	−0.5 (4)	C32—C31—C36—C35	1.2 (3)
C4—C5—C6—C7	0.1 (4)	C32—C33—C34—N2	−177.7 (2)
C5—C6—C7—C2	1.0 (4)	C32—C33—C34—C35	0.0 (3)
C5—C6—C7—C8	−179.3 (3)	C33—C34—C35—C36	0.5 (4)
C6—C7—C8—C9	0.7 (5)	C34—N2—C37—C38	−172.8 (2)
C6—C7—C8—C13	−179.2 (3)	C34—C35—C36—C31	−1.1 (4)
C7—C2—C3—C4	1.2 (4)	C36—C31—C32—C33	−0.7 (3)
C7—C8—C9—C10	−179.4 (3)	C37—N2—C34—C33	−139.8 (2)
C7—C8—C13—C1	−0.8 (3)	C37—N2—C34—C35	42.6 (3)
C7—C8—C13—C12	178.8 (2)	C37—C38—C39—O2	−6.4 (4)
C8—C9—C10—C11	0.4 (4)	C37—C38—C39—C40	171.8 (2)
C9—C8—C13—C1	179.2 (2)	C37—C38—C47—C42	−170.6 (2)
C9—C8—C13—C12	−1.1 (4)	C37—C38—C47—C46	8.2 (4)
C9—C10—C11—C12	−0.8 (4)	C38—C39—C40—C41	0.0 (4)
C10—C11—C12—C13	0.2 (4)	C39—C38—C47—C42	3.3 (4)
C11—C12—C13—C1	−179.7 (2)	C39—C38—C47—C46	−177.9 (2)
C11—C12—C13—C8	0.8 (4)	C39—C40—C41—C42	1.0 (4)
C13—C1—C2—C3	−178.8 (2)	C40—C41—C42—C43	−179.0 (3)
C13—C1—C2—C7	−0.4 (3)	C40—C41—C42—C47	0.2 (4)
C13—C1—C14—C15	−162.7 (2)	C41—C42—C43—C44	−179.4 (3)
C13—C1—C14—C19	21.7 (3)	C41—C42—C47—C38	−2.3 (4)
C13—C1—C31—C32	−110.1 (3)	C41—C42—C47—C46	178.8 (2)
C13—C1—C31—C36	71.6 (3)	C42—C43—C44—C45	0.1 (4)
C13—C8—C9—C10	0.5 (4)	C43—C42—C47—C38	176.9 (2)
C14—C1—C2—C3	−59.9 (3)	C43—C42—C47—C46	−2.0 (4)
C14—C1—C2—C7	118.5 (2)	C43—C44—C45—C46	−1.1 (4)
C14—C1—C13—C8	−114.0 (2)	C44—C45—C46—C47	0.4 (4)
C14—C1—C13—C12	66.4 (3)	C45—C46—C47—C38	−177.8 (2)
C14—C1—C31—C32	123.5 (2)	C45—C46—C47—C42	1.1 (4)
C14—C1—C31—C36	−54.7 (3)	C47—C38—C39—O2	179.7 (2)
C14—C15—C16—C17	1.7 (4)	C47—C38—C39—C40	−2.1 (4)
C15—C14—C19—C18	−2.0 (4)	C47—C42—C43—C44	1.4 (4)
C15—C16—C17—N1	−179.2 (2)		

### Hydrogen-bond geometry (Å, º)

**Table:**

*D*—H···*A*	*D*—H	H···*A*	*D*···*A*	*D*—H···*A*
O1—H1···N1	0.87 (3)	1.75 (3)	2.526 (3)	148 (3)
O2—H2···N2	1.00 (4)	1.63 (4)	2.558 (3)	152 (3)
C3—H3···O1^i^	0.95	2.58	3.461 (3)	155
C35—H35···O1^ii^	0.95	2.44	3.389 (3)	178
C28—H28···Cl1^iii^	0.95	2.86	3.770 (3)	161
C45—H45···Cl1^ii^	0.95	2.86	3.457 (3)	122
C46—H46···Cl1^ii^	0.95	2.88	3.465 (3)	121

Symmetry codes: (i) −*x*+1, −*y*+2, −*z*+1; (ii) −*x*+1, −*y*+1, −*z*+1; (iii) −*x*+2, −*y*+2, −*z*+1.
